# Nutritional solutions for appetite regulation in livestock and poultry: A review

**DOI:** 10.1016/j.aninu.2026.03.014

**Published:** 2026-08-21

**Authors:** Xiangyan Liu, Ruxin Yao, Shiyu Song, Chuan Zhou, Lixin Wen, Ji Wang

**Affiliations:** a Hunan Engineering Research Center of Livestock and Poultry Health Care, College of Veterinary Medicine, Hunan Agricultural University, Changsha 410128, Hunan, China; b Yuelushan Laboratory, Changsha 410128, Hunan, China; c Changsha Lvye Biotechnology Co., Ltd., Changsha 410100, Hunan, China

**Keywords:** Nutrition, Appetite regulation, Feed additives, Livestock, Poultry

## Abstract

The regulation of appetite in livestock and poultry is a complex process that involves the integration of central and peripheral physiological mechanisms. The process is regulated by nutritional, environmental, and genetic factors. This review synthesises current understanding of the neuroendocrine and gut**–**brain axis pathways that regulate appetite, including hypothalamic signalling via agouti-related protein (AgRP)/neuropeptide Y (NPY) and proopiomelanocortin (POMC)/cocaine- and amphetamine-regulated transcript (CART) neurons, as well as peripheral modulation by gut hormones (e.g., ghrelin, leptin [LEP], and glucagon-like peptide–1 [GLP-1]) and microbial metabolites (e.g., volatile fatty acids [VFA]). Distinct species–specific characteristics, such as the use of rumen fill and VFA of ruminants, visual feed preference in poultry, and taste–driven intake in swine, underline the need to develop proper nutritional strategies. The regulatory and potential mechanism of some important dietary factors, such as energy density, protein and amino acid balance, micronutrients and functional additives (e.g., phytochemicals, probiotics, and acidifiers), are discussed in this paper, which can enhance satiety or stimulate feed intake. Furthermore, the latest advancements in species- and stage-specific nutritional intervention strategies for swine, ruminants, and poultry are summarised. Emerging approaches, including novel alternative feed resources, multi-omics analysis, feed-focused genome editing, and precision feeding systems, offer promising strategies to improve feed efficiency and animal health. Future research should integrate molecular biology, nutrigenomics, and smart farming technologies to develop sustainable, species–specific strategies for appetite modulation, thereby enhancing livestock productivity and welfare.

## 1 Introduction

Appetite regulation in livestock and poultry is a pivotal determinant of animal performance, feed conversion efficiency, and economic benefits in animal husbandry. Feed intake directly determines nutrient intake, serving as the foundation for ensuring the full realisation of animals' genetic potential and production capacity. However, in practical production, feed intake is highly susceptible to a multitude of perturbations. Environmental and management factors (e.g., excessive stocking density, insufficient water supply, and poor feed palatability) (Adeniji et al., 2020; Amado et al., 2022; Averós et al., 2012; Kutay, 2024), physiological factors (e.g., stress and disease infection) (Collin et al., 2001; Ghulam et al., 2022; Merlot et al., 2016), and nutritional imbalances (e.g., low-protein diets, amino acid imbalance, trace element deficiency or excess, fatty acid imbalance, and inappropriate crude fibre content) (Khwatenge et al., 2019; Ramiah et al., 2020; Valent et al., 2020a) can alter feeding patterns and inhibit feeding behaviour through peripheral signals and changes in the excitability of central appetite–regulating nuclei.

In view of these findings, it is of great scientific and practical significance to explore nutritional intervention strategies to optimize the appetite regulation mechanism in livestock and poultry. Although the latest progress in genomics and precision nutrition provides opportunities for dietary customization based on physiological needs, there is still a gap in translating mechanistic insights into field applications. For instance, phytochemicals such as essential oils can enhance feed palatability and gut health, but their efficacy varies markedly among swine, ruminants, and poultry (Hundal et al., 2019; Roy et al., 2015; Zhao et al., 2024). This review links basic physiology with applied nutrition, including: (1) the central and peripheral physiological mechanisms of appetite regulation in livestock and poultry, including species-specific regulatory characteristics; (2) the mechanisms of key dietary nutritional factors (basic nutrients, micronutrients, and functional additives) regulating appetite through the gut–brain axis and hormonal pathways; (3) species–specific and stage–specific nutrition intervention strategies for regulating the appetite in swine, ruminants, and poultry; (4) the cutting–edge research direction and technical application of appetite regulation, covering the development of new feed resources, multi–omics analysis, gene editing and precise feeding systems. By integrating these views, this review aims to promote the transformation of appetite regulation research in a sustainable and accurate direction, and provide theoretical support for optimizing animal production performance in the context of the continuous growth of global demand for livestock products.

## 2 Physiological mechanisms of appetite control in livestock and poultry

The regulation of appetite in livestock and poultry is a complex physiological process that integrates signals from the central nervous system (particularly the hypothalamus) and peripheral sources including gastrointestinal hormones, adipose tissue, and metabolites.

### 2.1 Central appetite regulation

The central regulation of appetite is a complex physiological process that depends on the synergistic interactions of multiple hypothalamic regions, including the arcuate nucleus (ARC), ventromedial nucleus (VMN), dorsomedial nucleus (DMN), paraventricular nucleus (PVN), and suprachiasmatic nucleus (SCN) (Yi et al., 2017). Among these, the ARC is the core sensory center. Within the ARC, two functionally antagonistic neuronal populations–the orexigenic neuropeptide Y (NPY)/agouti–related protein (AgRP) neurons and the anorexigenic proopiomelanocortin (POMC)/cocaine- and amphetamine-regulated transcript (CART) neurons (Ando et al., 2001; Barb et al., 2006; Kuhla et al., 2011; Su et al., 2025; Szlis et al., 2018; Takeuchi et al., 2000) regulate feeding behaviour through projections to nuclei such as the paraventricular thalamus (PVT) and the lateral hypothalamic area (LHA) (Petrovich, 2021) (Fig. 1). While the anatomical framework of the hypothalamus is largely conserved across various livestock and poultry species, species-specific variations exist in neuropeptide expression, neural circuit functionality, and signalling pathway responsiveness.

**Fig. 1:**
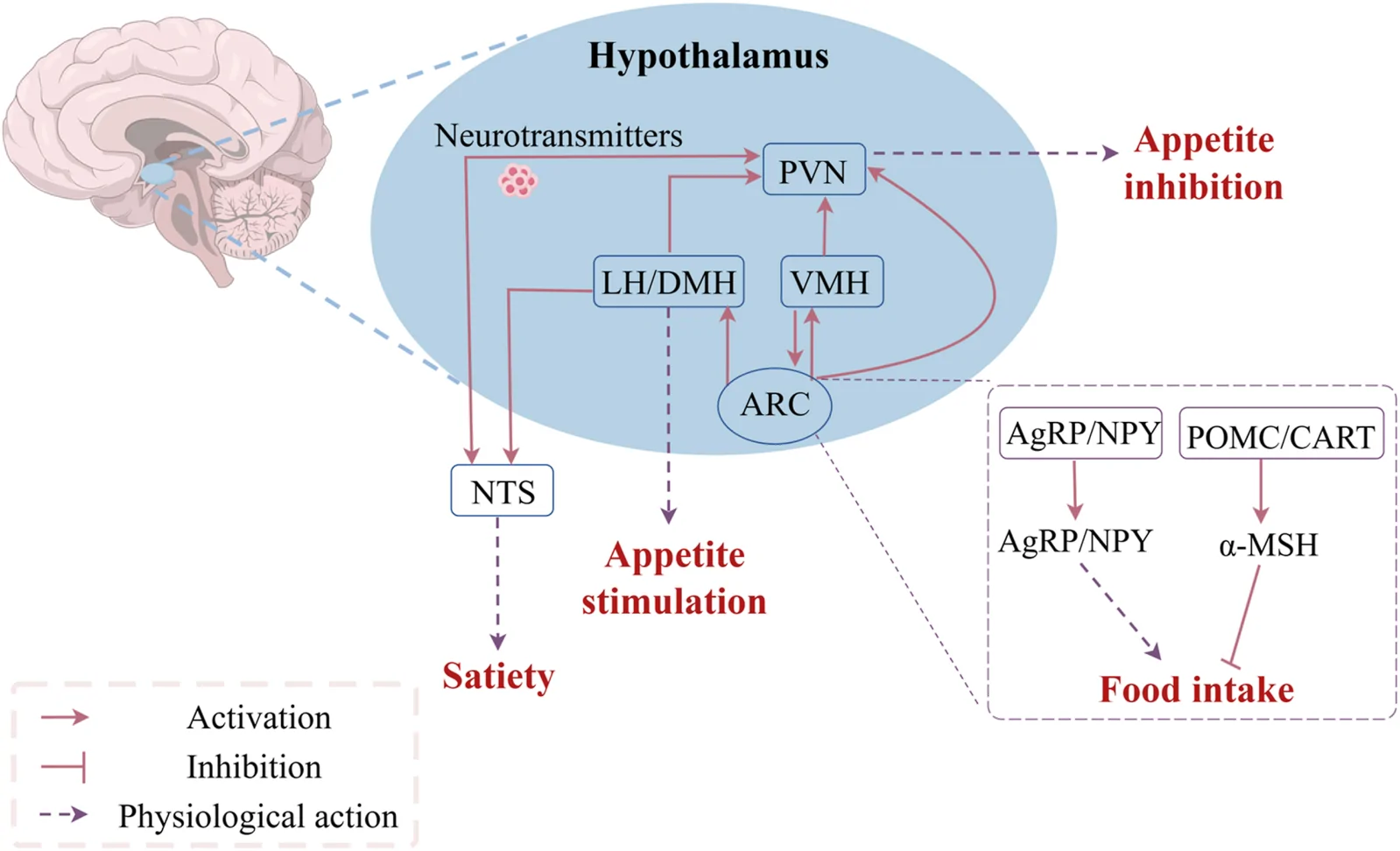
Hypothalamic nuclei and neuropeptide regulatory network. The ARC of the hypothalamus acts as a neural hub for appetite regulation, achieving dynamic balance through two classes of functionally antagonistic neurons: orexigenic neurons AgRP/NPY secrete NPY and AgRP to stimulate feeding behaviour; anorexigenic neurons POMC/CART secrete POMC, processed into α-MSH and CART to inhibit feeding. Signals from the ARC are transmitted to the PVN via the LH/DMH and VMH nuclei, ultimately leading to appetite inhibition through neurotransmitter release. Meanwhile, the NTS participates in satiety regulation after receiving peripheral signals, and the LH/DMH mediates the appetite-stimulation pathway. ARC = arcuate nucleus; AgRP = agouti-related protein; NPY = neuropeptide Y; POMC = proopiomelanocortin; CART = cocaine- and amphetamine-regulated transcript; α-MSH = alpha-melanocyte-stimulating hormone; PVN = paraventricular nucleus; LH = lateral hypothalamic; DMH = dorsomedial hypothalamic; VMH = ventromedial hypothalamic; NTS = nucleus of the solitary tract. This circuitry precisely regulates the feeding behaviour of livestock and poultry through complex interactions of activation (→), inhibition (⊣), and physiological actions (dashed lines).

Appetite regulation in swine is governed by a highly sophisticated hypothalamic energy-sensing network that closely parallels human physiology (Valent et al., 2020b). Furthermore, leptin (LEP) secreted by adipose tissue acts directly on the ARC to achieve a long-term balance between energy reserves and feed intake; the sensitivity of this feedback mechanism is very close to that of humans (Mármol-Sánchez et al., 2021).

In ruminants, central appetite regulation is not only mediated by neuropeptides, but also limited by their special digestive physiology and metabolic requirements. A distinguishing feature of their hypothalamic appetite control is that ruminal signals take precedence over metabolic signals, creating a significant divergence from that in monogastric animals. The PVN in ruminants exhibits markedly greater sensitivity to ruminal distension signals than that in monogastrics, reinforcing anorexigenic effects by releasing neuropeptides such as oxytocin (Kuhla et al., 2011). Furthermore, appetite regulation in ruminants relies on the central nervous system–melanocortin (CNS–MC) system. In sheep, Ehrhardt et al. showed that intracerebroventricular (ICV) administration of the α-MSH analog melanotan-I (MTI) activated central melanocortin signalling, leading to reduced feed intake and alterations in metabolic hormone profiles and insulin action, whereas inhibition of melanocortin signalling enhanced feeding behaviour (Ehrhardt et al., 2023).

Poultry exhibit a unique neuropeptide interaction pattern in appetite regulation and serve as the original species for the study of gonadotropin–inhibitory hormone (GnIH) (McConn et al., 2016). This neuropeptide exhibits a unique dual functionality in birds, characterised by an “increased feeding–suppressed reproduction” pattern. During energy restriction, the expression of GnIH in the poultry hypothalamus increases. On the one hand, it strongly promotes feeding via hypothalamic pathways (as in mammals), and on the other hand, it suppresses reproductive function through the hypothalamic–pituitary axis. This efficient energy reallocation pattern exists in birds, especially in seasonally breeding species (McConn et al., 2014, 2016; Song et al., 2024; Zhou et al., 2022). In contrast to its role as a potent orexigenic factor in mammals, orexin (also known as hypocretin) in chickens does not possess feeding–promoting functions (Furuse et al., 1999; Ohkubo et al., 2003). Its primary physiological roles are likely concentrated in maintaining arousal and regulating peripheral energy metabolism (in muscle and liver) (Ferver et al., 2020; Katayama et al., 2010).

In conclusion, while the hypothalamic structural framework is the same in these species, their regulating strategies are different in principle: the swine follow a classic mammalian energy balance regulation model, the ruminants prioritise physical gastric signals over metabolic feedback, and the poultry show neuropeptides that are significantly different from those in mammals. The specific regulation mechanism of these species represents an important evolutionary adaptation to their respective feeding ecosystems.

### 2.2 Central modulation by peripheral signals

The regulation of appetite by peripheral signals is also a complex and precise physiological process. It primarily involves the production of chemical signalling molecules (such as hormones and metabolites) by the gastrointestinal tract, adipose tissue, liver, and other organs. These molecules are transmitted to the ARC of the hypothalamus via blood circulation or neural afferent pathways, thereby dynamically regulating the body's energy intake (Chatterjee et al., 2023). While these physiological processes are relatively conserved across vertebrates, swine, ruminants, and poultry have evolved distinct species–specific variations in their peripheral regulatory mechanisms to adapt to divergent ecological niches, metabolic demands, and dietary patterns.

Appetite regulation in swine is highly similar to that of humans and rodents, with short–term satiety primarily mediated by gastrointestinal hormones such as cholecystokinin (CCK), glucagon–like peptide-1 (GLP-1), and peptide YY (PYY). At the same time, ghrelin serves as the key hunger signal driving feed intake behaviour (Fu et al., 2018). In long–term energy monitoring, adipose–derived LEP plays a central role (Tan et al., 2024). Genetic studies have demonstrated that variations in the LEP receptor gene influence feed intake behaviour and weight gain in swine, suggesting that the long–term regulation of porcine feeding behaviour by LEP signalling is genetically determined (Suárez-Mesa et al., 2023). It has been reported that ICV administration of 50 μg leptin significantly decreased feed intake in ovariectomized crossbred prepubertal gilts (Barb et al., 2006). Notably, recent metabolic studies have expanded upon this, GnIH exerts significant orexigenic effects in swine and induces hepatic gluconeogenesis and adipose tissue insulin resistance following 14–d intraperitoneal administration in female piglets, representing a novel discovery in the metabolic regulation of monogastric animals (Chen et al., 2022a; Stocks and Allen, 2014). Furthermore, microbial fermentation products are involved; Zhang et al. (2022) found that twice-daily cecal infusion of propionate (2 mmol/L, 25 mL) for 7 d via a simple T-type fistula stimulated colonic secretion of PYY and GLP-1, thereby reducing short–term feed intake.

In ruminants, appetite regulation is governed mainly by volatile fatty acids (VFA)—the products of ruminal fermentation–rather than the glucose–dominated patterns commonly observed in monogastric animals. Despite being inhibited by VFA, ghrelin still functions as a classical orexigenic factor. In Ile de France ewes, a single injection of 1 mg ovine ghrelin markedly elevated short-term feed intake, with values about 14 times higher than those observed in controls (Grouselle et al., 2008). Propionate is the primary satiety signal in ruminants; its hepatic oxidation transmits direct signals to the central nervous system to terminate feeding (Maldini et al., 2019). Continuous infusion of propionate at 0.5 mol/h for 18 h reduced dry matter intake (DMI) by 15% in Holstein dairy cows (Stocks and Allen, 2014). Moreover, ruminants show low insulin sensitivity, and their feeding behaviour is much less responsive to glucose fluctuations than that of monogastric animals, which is indeed a trait of their long–term adaptation to cellulose–based diets (Khare et al., 2023).

In the process of evolution, poultry has formed a very unique metabolic model, the function of ghrelin is opposite to that of mammals. In mammals, growth hormone-releasing peptide plays an appetite-promoting role through a unique growth hormone secretagogue receptor type 1a–cyclic adenosine monophosphate (GHS R1a-cAMP) pathway, while in poultry, it is an effective anorexia factor (Khan et al., 2014; Vizcarra et al., 2012). In broiler chicks, ICV administration of chicken ghrelin (0.5-2 μg) resulted in a suppression of feed intake (Saito et al., 2002). Recent studies have confirmed that liver–expressed antimicrobial peptide 2 (LEAP2) acts as a pivotal antagonist of the ghrelin receptor in poultry. Its levels fluctuate significantly with feeding status. Although fasting markedly downregulated LEAP2 expression in the liver and small intestine of broiler chicks, and refeeding restored its levels, direct investigations into the effect of LEAP2 on feed intake in poultry remain limited (Zheng et al., 2022). In addition, poultry exhibit significantly weakened or even functionally deficient LEP activity. Although an authentic *LEP* gene has been identified in poultry, with a sequence highly similar to that of the mammalian *LEP* gene (Taouis et al., 1998), its expression in adipose tissue remains extremely low. Moreover, circulating LEP levels do not consistently correlate with body fat deposition or feeding behaviour, rendering the hormone ineffective as a classic satiety signal (Ashwell et al., 1999). For instance, administration of mouse leptin to male broiler chicks and male Single Comb White Leghorn chicks did not affect feed intake at 2 or 24 h after injection (Bungo et al., 1999). Research indicates that avian LEP/leptin receptor (LEPR) likely operates via autocrine or paracrine signalling within localised tissues, such as the brain and pituitary gland, rather than regulating function through the classic adipocyte–hypothalamus endocrine axis (Seroussi et al., 2016). Moreover, intraperitoneal administration of CCK-8 in broiler chickens significantly reduced feed intake and delayed feeding behaviour. At a dose of 14 μg/kg, chicks developed an aversion to the feed color previously associated with CCK-8 injection, suggesting that CCK-mediated satiety signals can be linked to feed sensory cues via the vagus nerve, thereby influencing subsequent feeding behaviour (Covasa and Forbes, 1994). The PYY plays a different role in appetite regulation in poultry compared with mammals and may not be a major factor. Intravenous administration of chicken PYY at 1.5 nmol/kg body weight did not affect feed intake in male broiler chicks. However, doses of 3 or 6 nmol/kg body weight significantly reduced feed intake in a dose-dependent manner, possibly through Y2 receptors in the rostral brainstem to suppress feeding behaviour (Aoki et al., 2017). In another study, central administration of PYY at 59 or 118 pmol significantly increased feed intake within 2 h in broiler chicks (Ando et al., 2001).

Consequently, poultry appetite is driven primarily by the synergistic interplay of hypothalamic neuropeptides (e.g., orexin and NPY). It is more sensitive to short–term satiety signals, including gastric distension. Swine follow the classical mammalian paradigm, where appetite is primarily regulated by blood glucose levels and adipose tissue–derived signals (insulin and LEP). Ruminants, meanwhile, exhibit a distinct regulatory framework in which the hypothalamus integrates specialized satiety signals derived from rumen fermentation products.

### 2.3 Species-specific differences in appetite regulation among livestock and poultry

As a typical omnivorous species, pigs show significant behavioural plasticity in feeding patterns, which is regulated by olfactory and gustatory sensory inputs. As an omnivore with well-developed taste buds (about 15,000-20,000, compared with poultry, 24-400), swine show an obvious preference for sweet compounds, such as sucrose, and a strong dislike for bitter substances like enrofloxacin (reduced feed intake by 28.65%) (Gautier et al., 2020a; Liu et al., 2017). The feeding behaviour of ruminants is regulated by rumen filling and fermentation products (such as VFA) (Luan et al., 2024). Unlike monogastric species, ruminants demonstrate enhanced tolerance to high–fibre diets. However, excessive intake of non–structural carbohydrates, such as starch, can precipitate ruminal acidosis, reducing voluntary feed intake by dysregulating ruminal pH and microbial homeostasis (Chen et al., 2022b; He et al., 2022). Appetite in poultry is mainly driven by visual stimulation. Physical characteristics of feed, such as appearance, colour, and particle size, significantly stimulate their feeding desire. Broiler chickens preferentially consume larger feed particles, particularly those exceeding 1.18 mm and 2.36 mm in diameter, and only consume smaller particles or fine feed particles when larger particles become unavailable (Portella et al., 1988). In addition, during a 21–d observation period, broiler chickens demonstrated a stronger preference for uncolored feed, with intake being 29.2% higher than that of purple–colored feed (Vargas et al., 2025). Additionally, poultry appetite is also influenced by the photoperiod, with higher daily feed intake when kept under dark conditions (Yameen et al., 2020) (Fig. 2). Table 1 provides a comprehensive summary and extension of the major regulatory characteristics of swine, ruminants, and poultry.

**Fig. 2:**
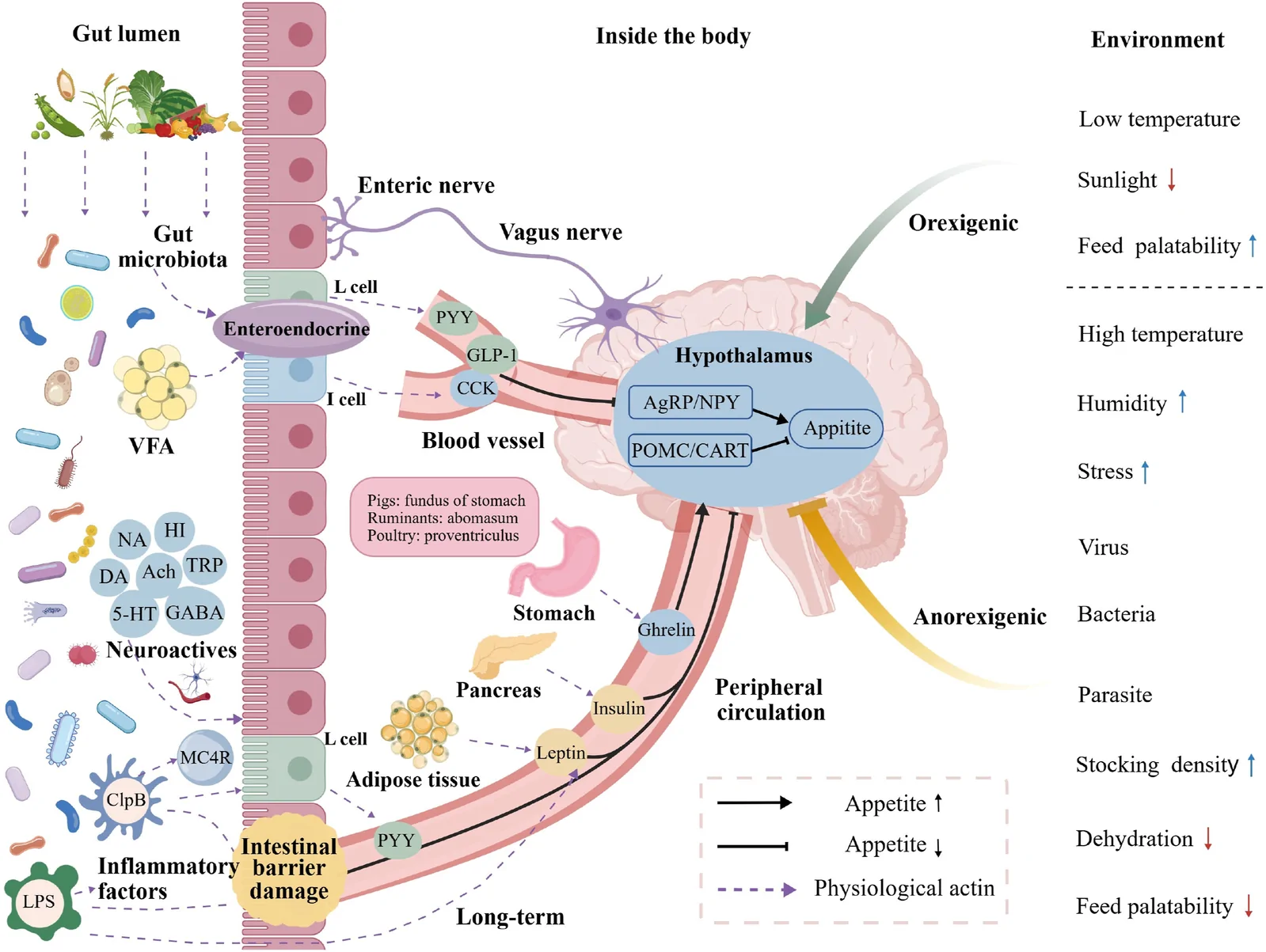
Integrated regulatory network of feed intake in livestock and poultry: Crosstalk among the gut–brain axis, peripheral signals, and environmental factors. Nutrients in the gut lumen interact with the gut microbiota, producing metabolites such as VFA and neuroactives (e.g., GABA, 5-HT, DA, NA, HI, TRP, and Ach). These signals stimulate enteroendocrine cells (L cells and I cells) to secrete anorexigenic hormones, including PYY, GLP-1, and CCK, which reach the hypothalamus via the peripheral circulation or the vagus nerve. The hypothalamus integrates signals from various organs: ghrelin from the stomach (e.g., pig fundus, ruminant abomasum, and poultry proventriculus) acts as an orexigenic signal; insulin from the pancreas and leptin from adipose tissue serve as long-term adiposity signals to inhibit appetite. Environmental factors such as low temperature, reduced sunlight, and high feed palatability stimulate appetite. In contrast, environmental stressors—including high temperature, high humidity, stress, viral/bacterial/parasitic infections, high stocking density, low water intake, and poor feed palatability—suppress appetite. Furthermore, LPS triggers systemic inflammation that chronically modulates leptin levels, thereby inhibiting feeding. Additionally, ClpB acts as an α-MSH mimetic that activates the MC4R and stimulates PYY production, thereby suppressing appetite. VFA = volatile fatty acids; GABA = gamma-aminobutyric acid; 5-HT = 5-hydroxytryptamine; DA = dopamine; NA = noradrenaline; HI = histamine; TRP = tryptophan; Ach = acetylcholine; PYY = peptide YY; GLP-1 = glucagon-like peptide-1; CCK = cholecystokinin; AgRP = agouti-related protein; NPY = neuropeptide Y; POMC = proopiomelanocortin; CART = cocaine and amphetamine regulated transcript; LPS = lipopolysaccharides; ClpB = caseinolytic protease B; α-MSH = alpha-melanocyte-stimulating hormone; MC4R = melanocortin 4 receptor. The circuitry precisely regulates the feeding behaviour through complex interactions of activation (→), inhibition (⊣), and physiological actions (dashed lines).

**Table 1:** Comparative table of key appetite mechanisms in livestock and poultry.

Features	Swine	Ruminants	Poultry
Feeding drive	They are highly dependent on taste and smell, showing a preference for sweet tastes and an aversion to bitter tastes, and their intake is strongly influenced by palatability. A high-fat sucrose Western-type diet increased feed intake in yucatan minipigs, while high-sucrose feed (20%) was preferred over low-sucrose feed (0%), with intakes of 433.5 ± 28.4 g vs. 108.3 ± 16.8 g (Gautier et al., 2020). Feeding diets containing free enrofloxacin reduced feed intake in pigs by 28.65% (Liu et al., 2017).	They are primarily governed by oral tactile sensation and post-ingestive feedback, with feed intake regulated through the integration of pre-ingestive sensory perception and physiological responses after ingestion. Negative energy balance (e.g., – 43.5 MJ retained/d in early lactation vs. + 7.2 MJ retained/d in late lactation) stimulates preprandial ghrelin secretion and GH pulsatility in lactating dairy cows (Bradford and Allen, 2008).	They exhibit high sensitivity to feed colour, form, and particle size. Broiler chickens preferentially select larger feed particles, particularly those exceeding 1.18 and 2.36 mm in diameter (Portella et al., 1988). Additionally, during a 21-d observation period, Cobb broilers demonstrated a stronger preference for uncolored feed, with intake being 29.2% higher than that of purple-colored feed (Vargas et al., 2025).
Satiety signal	Gastric distension and gut hormones play a key role. High lysine intake suppresses feed intake in weaned piglets by enhancing jejunal CCK secretion, indicating a CCK-mediated anorexigenic effect (Müller et al., 2024). Time-restricted feeding (ad libitum access 07: 00-19: 00) reduced feed intake and increased GLP-1 secretion in male Duroc × Landrace × Large white pigs (Li et al., 2025).	Rumen fill and distension serve as the strongest satiety signals. The VFA particularly propionate, function as key chemical satiety signals. Continuous infusion of propionate at 0.5 mol/h for 18 h reduced DMI by 15% in Holstein dairy cows (Stocks and Allen, 2014). In lactating cows, intraruminal infusion of 2–8 mol sodium propionate depressed DMI in a dose-dependent fashion, with each 1 mol of propionate corresponding to a drop of roughly 140 g DMI (Anil et al., 1993)	Crop and gizzard distension act as unique physical signals, whereas ghrelin, LEAP2, and intestinal hormones (e.g., CCK) serve as chemical satiety signals (Khan et al., 2014; Zheng et al., 2022). The ICV injection of chicken ghrelin at 0.5–2 μg inhibited food intake in broiler chicks (Saito et al., 2002). Fasting markedly reduced LEAP2 expression in the liver and small intestine of broiler chicks, whereas refeeding restored its levels (Zheng et al., 2022). The CCK inhibits feed intake via vagus nerve-mediated gut–brain signaling and participates in post-ingestive feedback regulation. The CCK-8 injection at 14 μg/kg suppressed food intake and delayed feeding behavior in broiler chickens (Covasa and Forbes, 1994).
Key hormone	Ghrelin, which promotes appetite, is produced in the gastric fundus (Fu et al., 2018); leptin is associated with adipose tissue deposition, but its regulatory effects are less sensitive than those observed in rodents (Mármol-Sánchez et al., 2021); ICV injection of leptin at 50 μg suppressed food intake in OVX crossbred prepubertal gilts (Barb et al., 2006).	Ghrelin is suppressed by VFA and is produced in the abomasum (Fukumori et al., 2012); Administration of 1 mg ovine ghrelin markedly increased short-term feed intake in Ile de France ewes, resulting in an approximately 14-fold increase compared with controls (Grouselle et al., 2008). Leptin primarily reflects body condition and energy balance. Intravenous injection of recombinant leptin at 0.2 μg/kg in heifers did not produce a significant increase in cerebrospinal fluid leptin concentrations (Williams et al., 2023).	Ghrelin, which suppresses appetite, is produced in the proventriculus (Khan et al., 2014); leptin function is attenuated. In male broiler chicks and male Single Comb white Leghorn chicks, injection of mouse leptin did not affect feed intake within 2 or 24 h (Bungo et al., 1999); The expression and functional role of PYY differ from those observed in mammals, and its anorexigenic effects appear to be inconsistent. In broiler chicks, intravenous chicken PYY at 1.5 nmol/kg BW did not affect feed intake, whereas doses of 3 or 6 nmol/kg BW decreased it dose-dependently (Aoki et al., 2017). By contrast, central delivery of PYY at 59 or 118 pmol markedly raised feed intake over a 2-h period (Ando et al., 2001).
Characteristics of central regulation	The functions of the hypothalamic AgRP/NPY and POMC/CART systems are conserved (Valent et al., 2020). The ICV injection of NPY significantly increased cumulative feed intake in ovariectomized prepubertal gilts (Barb et al., 2006). Swine possess a strong food reward regulatory system. Exon sequencing of 201 taste- and appetite-reward-related genes across 304 pigs of diverse breeds uncovered numerous genetic variants with putative effects on protein function, pointing to a role for genetically governed feed perception and reward pathways in modulating feeding behavior (Clop et al., 2016).	Depends on metabolic feedback and hypothalamic integrative regulation. The hypothalamus exhibits dual sensitivity to VFA (Baile and McLaughlin, 1970) and blood glucose levels (Mühlhäusler et al., 2005). In fetuses of Merino ewes, glucose injection upregulated *POMC* mRNA expression (Mühlhäusler et al., 2005). Administration of NPY at 0.3 μg/kg to castrated male sheep promoted feed intake and restored the anorexic response induced by lipopolysaccharide (McMahon et al., 1999).	The structure of the hypothalamic appetite centres is conserved. It responds sensitively to photoperiod, thereby indirectly regulating feeding rhythm (Olanrewaju et al., 2019). Compared with the short/non-intermittent photoperiod (8L: 16D), the regular/intermittent photoperiod (2L: 2D) increased feed intake by 3.38% in broilers (Olanrewaju et al., 2019).

CCK = cholecystokinin; GLP-1 = glucagon-like peptide-1; DMI = dry matter intake; GH = growth hormone; LEAP2 = liver–expressed antimicrobial peptide 2; BW = body weight; PYY = peptide YY; VFA = volatile fatty acids; AgRP = agouti-related protein; NPY = neuropeptide Y; POMC = proopiomelanocortin; CART = cocaine- and amphetamine-regulated transcript; ICV = intracerebroventricular; OVX = ovariectomized.

## 3 Mechanisms of key nutritional regulation factors in livestock and poultry

Appetite regulation in livestock and poultry is profoundly influenced by dietary composition. Specifically, energy, macronutrients, micronutrients, and bioactive substances precisely regulate appetite by mediating the gut**–**brain signalling axis and hormonal pathways, with their underlying mechanisms exhibiting both cross–species commonalities and species–specific characteristics.

### 3.1 Basic nutritional factors: regulatory effects of energy and proteins/amino acids

As core nutritional substrates, energy and proteins/amino acids maintain appetite homeostasis by regulating the central adenosine monophosphate–activated protein kinase (AMPK) pathway and intestinal hormone secretion (Fan et al., 2025b; Stark et al., 2013). Among these regulatory pathways, the glucose–insulin–GLP–1 axis is a conserved cross–species signalling cascade (Brüning and Fenselau, 2023). In contrast, the specific regulatory patterns exhibit distinct species–specific characteristics owing to differences in digestive system structures and nutrient–sensing mechanisms of livestock and poultry. In terms of energy regulation, pigs and poultry are highly sensitive to glucose and fat in the diet (Kidd et al., 2021a; Xu et al., 2022). Glucose can activate intestinal L–cells to secrete GLP-1 through the intestinal sodium–glucose cotransporter 1 (SGLT1), thereby generating dose–dependent satiety feedback (Moran et al., 2010). The liver of poultry serves as a core sensor of energy status, adjusting feed intake in response to dietary energy concentration. However, the existing research results on the effect of a high–energy diet on chicken feed intake are still controversial. Mao et al. (2024) reported that high–energy and high–amino acid diets (basal diet + 100 kcal/kg metabolizable energy [ME] + 15% digestible amino acids) could alleviate the reduction in feed intake in yellow–feathered broilers caused by heat stress. The average daily feed intake (ADFI) of yellow–feathered broilers fed the basic control diet was 171.52 ± 15.64 g, while the ADFI of yellow feather broiler chickens treated with high–energy and high–amino acid daily ration is 147.84 ± 15.53 g, a significant decrease of 13.81%. In contrast, studies have shown that dietary energy levels have no significant effect on feed intake in Hy-Line chicks (Choi et al., 2012). Meanwhile, Rozenboim et al. (2016) reported that only the group fed a low–protein, high–energy diet exhibited a marked decrease in appetite. In this study, 15–wk–old White Leghorn (WL), Hy–line pullets were used. Compared with normal protein, normal fat, normal ME group (17.5% crude protein [CP] + 3.5% fat + 2750 kcal/kg), normal protein, high fat, high ME group (17.5% CP + 7% Fat + 3000 kcal/kg), and low protein, normal fat, normal ME group (13% CP + 3.5% Fat + 2750 kcal/kg), pullets fed the low protein, high fat, high ME group (13% CP + 6.2% Fat + 3000 kcal/kg) showed a significant 20%–27% decline in ADFI. Collectively, these results indicate that changes in energy levels alone have a limited effect on poultry feed intake. For ruminants, blood sugar does not regulate feed intake; On the contrary, they mainly rely on VFA and amino acids produced by rumen microbial fermentation to meet their energy and nitrogen requirements (Urga et al., 2025). The VFA, including acetate, propionate, and butyrate, are not only a primary source of energy but also can activate G protein-coupled receptor (GPR)41/43 in rumen epithelial cells, which transmits satiety signals to the hypothalamus through the vagus nerve (Wang et al., 2009). The plasma concentrations of these VFA directly influence rumen motility and feed intake (Gaspe et al., 2025; Toledo et al., 2023). Experiments in different ruminant breeds have verified this mechanism. In lactating cows, intraruminal infusion of 2 to 8 mol sodium propionate depressed DMI in a dose-dependent fashion, with each 1 mol of propionate corresponding to a drop of roughly 140 g DMI (Anil et al., 1993). In neonatal lambs, daily intragastric administration of sodium butyrate at 0.36 g/kg body weight significantly improved feeding performance: ADFI increased from 103.39 ± 3.47 g/d to 118.29 ± 2.08 g/d, and average daily gain (ADG) rose from 103.39 ± 3.47 g/d to 118.29 ± 2.08 g/d. Ruminal total VFA concentration also increased markedly (Liu et al., 2019).

In terms of protein/amino acid regulation, reducing dietary protein levels can mitigate the adverse effects of heat stress on animal production performance (Sutton et al., 2024). The cross–species adaptability of the tryptophan–serotonin pathway has been verified by both in vitro and in vivo experiments, and tryptophan supplementation can effectively improve the feeding performance of pigs and poultry under pathological conditions (Fan et al., 2025a,b). Nevertheless, existing studies show clear discrepancies: the regulatory effect of leucine on appetite varies across species and with dose. Increased dietary leucine levels (ratio 150) impair Lohman Indian River broiler growth performance (Kidd et al., 2021b). However, in pigs, leucine demonstrates a differential effect characterised by “low–dose promotion and high–dose inhibition”. Kwon et al. (2022) formulated diets with three levels of dietary leucine (101%, 200%, or 299% standardized ileal digestible [SID] Leu: Lys) in a 3 × 3 factorial design over 21 d. Increasing dietary leucine from 101% to 299% SID Leu: Lys significantly reduced ADG (777 g/d vs. 852 g/d in the control) and ADFI (1506 g/d vs. 1630 g/d in the control) in growing pigs (Line 359 × Camborough cross pigs). In finishing pigs (Duroc × Landrace × Large cross pigs), Yin et al. (2024) reported that dietary leucine supplementation at 0.25% significantly increased ADG (0.61 ± 0.03 g/d vs. 0.42 ± 0.03 g/d in the control) and ADFI (2.46 ± 0.12 g/d vs. 2.26 ± 0.07 g/d in the control). A comparison of these two sets of experiments reveals that such discrepancies stem from differences in dietary energy–to–protein ratios and experimental durations. It should be noted that poultry are particularly sensitive to amino acid imbalances, and they need tightly balanced formulations. Studies have shown that reducing the dietary level of essential amino acids such as lysine, methionine, threonine, and tryptophan will significantly reduce the feed intake of broilers (Cordero et al., 2025). At the same time, excessive supplementation of tryptophan, lysine, methionine, threonine, and histidine can also reduce the appetite of broilers and inhibit weight gain (Acar et al., 2001). In pigs, oral lysine, isoleucine, and leucine have an anorexigenic effect via CCK, while glutamic acid and phenylalanine enhance insulin secretion by increasing the level of circulating GLP-1 (Müller et al., 2023). The amino acid sensing ability of ruminants is limited, and the protein level of daily food mainly regulates the rumen microbiota and indirectly regulates appetite.

In summary, energy and protein/amino acids are fundamental levers to control appetite, but as mentioned above, the use of these levers requires the accuracy given by species–specific physiology. Swine and poultry are highly sensitive to the density of energy in the diet and to the form of the carbohydrate to use the insulin/GLP-1 pathway for satiety. Poultry are particularly sensitive to the imbalances of amino acids, and for them the formulations have to be tightly balanced. Ruminants work on a different principle, where appetite is mostly a readout of the health of the rumen fermentation, especially VFA, rather than glucose or a single amino acid.

### 3.2 Micronutrients: mediating effects of vitamins and minerals

Micronutrients affect appetite via different physiological pathways in different animal species. Swine and poultry are more dependent on these nutrients to preserve intestinal integrity and unimpeded hormonal signalling. In contrast, ruminants rely primarily on micronutrients to support rumen microbial community stability and the generation of fermentation products. Among existing research, the regulatory mechanisms of zinc, B vitamins, and vitamin D are relatively well–documented, with strong cross–species commonality. Zinc can maintain the integrity of intestinal tight junction proteins, such as zonula occludens-1 (ZO-1), and improve livestock growth performance (Huang et al., 2019; Yang et al., 2024b). Especially in pigs, the appetite-regulating effect of zinc is significant, and its mechanism has been relatively well elucidated. Zinc deficiency not only reduces the rate of taste bud renewal and impairs the sensory drive for feeding, but also directly interferes with hypothalamic LEP signal transduction. In DNA 200 × 400 weaned piglets (initial body weight: 6.0 ± 0.08 kg), Dahmer et al. (2024) evaluated five dietary treatments over 42 d, including a negative control (110 ppm Zn from zinc sulfate), Low–zinc oxide (ZnO) (400 ppm added ZnO), High–ZnO (3000 ppm ZnO in phase 1 and 2000 ppm in phase 2), and corresponding microencapsulated ZnO treatments. During the common phase 3 (d 28–42), pigs fed the negative control or microencapsulated ZnO had improved ADG and ADFI compared to those fed High–or Low–ZnO. In a 14–d post-weaning study, Ng'ang'a et al. (2025) reported that piglets fed 3000 mg/kg ZnO had higher ADG (165 g/d vs. 123 g/d in the control), tended to have increased ADFI (204 g/d vs. 181 g/d in the control), and showed improved gain–to–feed ratio (0.669 vs. 0.774 in the control) compared to controls. Furthermore, Long et al. (2023) demonstrated that dietary supplementation with 500 mg/kg porous or nano ZnO particles increased ADFI (d 1–28) and ADG compared to the basal diet group. There is an obvious dose–response relationship exists between zinc supplementation dose and improvements in appetite (Cai et al., 2025; Dahmer et al., 2024; Yin et al., 2009). The regulatory effect of selenium is species–dependent. In ruminants, selenium can alleviate rumen inflammation and improve appetite (Brady et al., 2025). Wang et al. (2023) demonstrated that dietary supplementation with 4.8 mg/kg selenium–yeast can enhance antioxidant and immune functions by improving muscle immunity and reducing the concentrations of inflammatory molecules in growing goats. In addition, the regulatory effect of B vitamins is the most unique and critical in ruminants. Traditionally, it is believed that ruminants are not prone to lack B vitamins, because rumen microorganisms can synthesize most B vitamins. However, under high–yield or high–stress conditions, the host's demand for vitamins, such as thiamine (vitamin B_1_), may exceed the synthetic capacity of the rumen microbiota (Ma et al., 2022). Due to their extremely high growth rate and unique calcium metabolism requirements, poultry are more sensitive to vitamin D_3_ deficiency. Severe vitamin D_3_ deficiency not only leads to skeletal problems but also causes systemic metabolic disorders and significant appetite reduction (Gao et al., 2022; Han et al., 2023). In 1–d–old Arbor Acres broilers, Han et al. (2023) demonstrated that dietary vitamin D_3_ supplementation ranging from 125 to 2000 IU/kg significantly increased body weight gain (BWG), feed intake, bone weight, and the percentage and weight of calcium and phosphorus in the tibia and femur compared with the negative control diet (0 IU/kg vitamin D_3_). Notably, supplementation with 125 IU/kg vitamin D_3_ alone increased BWG and feed intake compared with the unsupplemented controls, and raising dietary vitamin D_3_ levels from 125 to 1000 IU/kg further enhanced BWG, feed intake, and bone mineralization, indicating a clear dose-dependent response.

In summary, most current studies rely on single–nutrient deficiency models, which are disconnected from the complex nutritional environment in actual production. Moreover, research on the synergistic and antagonistic effects between micronutrients remains limited. For instance, an imbalance in the zinc–copper ratio can counteract the protective effect of zinc on the intestinal barrier (Marchetti et al., 2025), a problem that has not received sufficient attention in cross–species applications.

### 3.3 Functional nutritional factors: synergistic regulatory effects of additives

Functional regulatory factors (including probiotics/prebiotics, acidifiers, and plant–derived feed additives) regulate appetite by synergising with nutrient metabolism. In the monogastric environment of pigs, specific strains such as *Lactobacillus* and *Bacillus* primarily act via the “metabolite**–**gut**–**brain axis” (Sun et al., 2022). Indole–3–lactic acid, a metabolite produced by *Lactobacillus*, can promote GLP-1 secretion and enhance satiety signal transmission via vagal afferent nerves (Li et al., 2025). Meanwhile, these strains maintain intestinal barrier integrity through competitive exclusion, reducing systemic inflammation induced by endotoxin translocation, a well–established inhibitor of appetite (Jiang et al., 2023; Li et al., 2024a; Tang et al., 2024). Poultry have a short digestive tract with rapid digesta transit, so the effects of probiotics and prebiotics are more focused on foregut colonisation and nutrient predigestion (Beyari et al., 2024; Veluri et al., 2024). For example, the colonisation of *Lactobacillus* in the crop and gizzard can initiate the hydrolysis of starch and protein in advance, increasing nutrient absorption rates and helping stabilise blood glucose levels, thereby reducing compensatory overfeeding caused by delayed nutrient absorption (De Cesare et al., 2020). Probiotic intervention in ruminants is entirely focused on “rumen microbiota modulation”. Direct**-**fed microbials (e.g., *Saccharomyces cerevisiae*) stabilise rumen pH by providing organic acids and vitamins, thereby improving feed intake and feed efficiency in fattening calves (Xiao et al., 2016). In a study by Maamouri and Ben Salem (2022), 16 Holstein calves were randomly assigned to a control group (wheat straw 5 kg DMI/d + concentrate 8 kg DMI/d) or a live yeast group receiving an additional 28 g/calf per d of *Saccharomyces cerevisiae* powder on the concentrate. Supplementation significantly improved ADG by 400 g/calf and final BWG by 39.1 kg/calf, while feed conversion rate (FCR) was significantly reduced.

Furthermore, acidifiers have been demonstrated to optimise the intestinal environment, enhance nutrient sensing, and increase feed intake in swine and poultry (Dahmer et al., 2022; Dong et al., 2024). In DNA Line 200 × 400 pigs, dietary supplementation with 0.5% benzoic acid over 28 d significantly increased body weight (21.4 vs. 20.9 kg in the control), ADG (398 vs. 378 g/d in the control), and ADFI (548 vs. 527 g/d in the control) (Warner et al., 2023). In addition, supplementation with 1 g/kg sodium diformate (NaDF) over 38 d in 1-d-old Arbor Acres broiler chickens increased body weight by 3% and ADFI by 2% (Sun et al., 2024). However, in ruminants, acidifiers may disrupt the balance of rumen fermentation, leading to appetite fluctuations (Cao et al., 2020). Rathert-Williams et al. (2021) reported that supplementation with calcium propionate at 100 or 300 g/d significantly decreased DMI compared with the control group in Holstein steers fed a finishing diet. Additionally, it is worth noting that plant–derived additives are considered safe at recommended dosages, with almost no residue issues in animal products (Bampidis et al., 2023). Nevertheless, systematic and comprehensive toxicological studies remain insufficient (Cheng et al., 2014). For example, some studies have shown that high tannin content in plant–derived feed additives can negatively affect appetite or feed utilisation efficiency (Faehnrich et al., 2016; Provenza et al., 2000). At high doses (47.06 g/kg), wormwood additives can significantly reduce broiler chicks' feed intake (4694.96 vs. 4670.48 g in the control) (Cetin et al., 2019).

In summary, functional additives modulate appetite by targeting specific nodes within the gut**–**brain axis, but their efficacy and mechanism are profoundly species–specific. The future is to find mechanism–defined additive combinations that will support synergistic gut health, microbial metabolism and hormone release.

## 4 Nutritional intervention strategies for appetite regulation

Growth performance is considered a core parameter in the management of animal husbandry in intensive livestock production systems. Accurate nutrition has become a key technical method to optimize the economic efficiency of animal husbandry by dynamically adapting to physiological conditions, improving feed conversion efficiency and meeting metabolic requirements.

### 4.1 Nutritional strategies for appetite modulation in swine

In swine production systems, different stages of development have differentiated nutritional needs and metabolic characteristics, so it is necessary to formulate precise nutritional management strategies for specific stages. Research has demonstrated that sow–piglets exhibit characteristic physiological features, including immature digestive system development, natural taste preference for lactose, and compromised intestinal barrier function. The main aetiological factors for the feeding crisis in this period are the nutritional composition difference and palatability difference between the maternal milk and the creep feed, and the poor digestive enzyme activity (amylase and protease) and the unstable establishment of gut microbiota (Bailey et al., 1956; Hampson and Kidder, 1986; Xun et al., 2023). In the creep feeding stage, insufficient feeding may lead to a decrease in weaning weight, an increase in stress sensitivity after post–weaning, and ultimately damage later growth performance. On the contrary, sufficient supplementary feeding has been proven to effectively promote the increase of food harvest after post–weaning (Bruininx et al., 2002). Furthermore, supplementation of creep feed with 0.1% feed flavour enhancers, such as cheese aroma compounds, increased pre-weaning feed intake by approximately 23%, and this effect persistently influenced post–weaning feed intake, with the cheese flavour group showing an increase of about 11.4% (Yan et al., 2011).

Feeding crisis is one of the critical transition phases in growth stages, and weaning is the most representative. The decrease in feed intake in this period is mainly a result of combined stressors, including environmental, social, and physiological stressors, which suppress feeding behaviour through the peripherical signalling pathways such as intestinal inflammatory cytokines and abnormal hormone secretion (Campbell et al., 2013). Inadequate feed intake during this phase may lead to post–weaning diarrhoea, growth stagnation, and prolonged recovery periods. It was found that a reduction in feed intake in the early post–weaning period directly causes growth retardation and intestinal dysfunction of the small intestine with villous atrophy and an increase in harmful fermentation metabolites. This insufficient intake can also cause intestinal inflammation, which is a negative feedback (Spreeuwenberg et al., 2001). Notably, a key study found that feeding weaned piglets with extruded feed composed of corn and broken rice reduced feed intake, with reductions of 55.5 and 71.5 g/d, which was related to the increase in plasma levels of GLP-1 and PYY (Zhuo et al., 2022). The ZnO is widely used to improve the feed intake of weaned piglets by stimulating the secretion of ghrelin (Dahmer et al., 2024; Yin et al., 2009). However, due to the risk of metal pollution to the environment, many countries, including China, have imposed stricter regulations on its application (Portiannyk et al., 2022). This regulatory shift has redirected research interest to safer alternatives, such as enzyme preparations and organic acid formulations. Studies indicate that supplementing weaned piglet diets with 0.05% α-amylase and lipase not only enhances feed intake during the first 30 d after weaning, but also significantly improves ADFI (599.61 vs. 528.82 g/d in the control) and ADG (344 vs. 258 g/d in the control) (Portillo et al., 2021). Therefore, the nutritional intervention at this critical stage focuses on relieving stress, repairing intestines, and regulating appetite.

In the breeding management of growing pigs, heat stress is the core environmental factor that limits the amount of food. Due to the high growth potential of modern pig breeds after selection, their metabolic heat production has increased significantly, and the burden of body temperature regulation has increased, so they are more sensitive to heat stress (Brown-Brandl et al., 2001). For example, when the ambient temperature is maintained at 32 °C, the voluntary feed intake of crossbred barrows and gilts decreases significantly, by 9.7% (Mills et al., 2024). In addition to responding to the challenges of specific production stages, technological innovation that can achieve precise nutrition supply has become a key path to improve the overall feed efficiency and health level of pigs. The precise feeding system can dynamically adjust the ratio of amino acids in daily rations according to individual weight, feeding behaviour, and growth rate, to optimize the efficiency of nutritional utilization (Santos et al., 2018). Research shows that such technologies not only enhance nutrient utilisation efficiency, but also reduce lysine intake by 26%, nitrogen excretion by 30%, and reduce the cost of raising per pig by $7.6 (Andretta et al., 2014). In addition, although the content of plant fibre in the diet is usually negatively related to the growth rate, adding 10% of corn silage to the pig diet can increase cumulative feed intake by 25.3%. This strategy shows good application prospects and economic benefits in commercial pig production (Lyu et al., 2022).

The physiological and metabolic characteristics of pigs at different development stages determine their different nutritional requirements. During the lactation and weaning periods, challenges in feed intake frequently compromise intestinal health and subsequent growth performance. Intervention measures such as flavour regulation, enzyme preparation application, and precise nutrition regulation can effectively alleviate stress response, increase feed intake, and promote intestinal development.

### 4.2 Nutritional strategies for appetite modulation in ruminants

Among ruminants (represented by dairy cattle and meat sheep), the nutritional requirements are mainly about the balance of energy, protein, and trace elements. Influenced by their unique digestive physiology, ruminants need more dietary fibre content and digestibility. Nutritional requirements vary according to physiological stages (e.g., pregnancy, lactation, and dry milk stage) and production goals (e.g., weight gain, milk yield, and meat quality improvement). Feed intake fluctuates considerably during critical phases such as the periparturient period and rumen development stage, and this fluctuation significantly impacts overall production performance (Berends, 2014; Feyissa et al., 2015). The periparturient period is generally regarded as a decisive “make–or–break” phase that determines lactation performance throughout the cycle. However, this period is commonly accompanied by a significant decrease in DMI. This hypophagia is driven not only by the physical reduction in rumen capacity during fetal development, but also by elevated circulating non-esterified fatty acids (NEFAs), which inhibit appetite via metabolic signalling pathways (Kuhla et al., 2016). Further research shows that incomplete oxidation of excess NEFAs results in systemic accumulation of ketone bodies, thus aggravating appetite suppression (Kang et al., 2025). Therefore, vitamin B_12_ supplementation can enhance the gluconeogenesis of rumen propionate, effectively prevent energy deficiency related to ketosis, and mitigate the hypophagic responses (Girard and Duplessis, 2023). Supplementation with rumen-protected commercial choline (15.0 g/d) and methionine (15.0 g/d) increased postpartum DMI by 1.0 to 2.3 kg/d and significantly reduced plasma β-hydroxybutyrate (BHBA) concentrations, thereby alleviating negative energy balance in periparturient dairy cows (Sun et al., 2016).

In addition to dietary modifications, the optimization of feeding management strategy is also an important way to improve perinatal appetite. Based on the evolutionary feeding behaviour characteristics of ruminants, night feeding (21:00) resulted in a 43.4% higher DMI within the first 3 h post–feeding compared to the morning feeding group (09:00) (Nikkhah, 2013, 2014). Compared with dairy cattle, meat sheep have smaller rumen capacity and are more sensitive to forage palatability; for this reason, the interventions have to be focused on forage optimisation and body condition balance. For example, dietary supplementation with sodium bicarbonate (15 g/kg DMI) effectively increased DMI in goats (by 31%) and sheep (by approximately 30%), demonstrating consistent promoting effects under heat stress and normal conditions (Jamal et al., 2021; Tripathi et al., 2004).

Beyond mineral supplementation, the characteristics of dietary fibre itself have also proved to be effective. Research indicates that bio–fermented straw significantly improves, when supplemented at 40%, significantly increases DMI and improves digestibility by 10.9% in sheep, thus improving growth performance and feed utilization (Kyawt et al., 2024). Although physiological feed intake depression is generally absent in calves and lambs, intensive production systems still necessitate interventions to maximise productivity. Dietary supplementation with 15% *Leucaena leucocephala* leaf meal in fattening lambs has been shown to moderately increase feed intake (by 10.8%), while at 75% inclusion, it significantly increases the digestibility of neutral detergent fiber (by 27.3%) (Ardeshiri et al., 2022). A 0.2% Chinese herbal complex additive (CCHM) has been shown to significantly increase ADFI in lambs by 20.29% (Wang et al., 2025).

### 4.3 Nutritional strategies for appetite modulation in poultry

Poultry species (represented by broilers and laying hens) show unique physiological characteristics, including high metabolic rates, short gastrointestinal tracts, rapid digesta transit, and high sensitivity to environmental stress (Francoeur et al., 2025; Guo et al., 2022). Their feed intake regulation mechanisms are significantly different from those of swine and ruminants, among which the impact of environmental factors like heat stress and stocking density has a disproportionately greater impact on poultry feed intake (Nanto-Hara et al., 2023). In modern poultry production systems, heat stress is the main and unavoidable factor leading to the decline in the feed intake of broilers. To reduce feed–induced heat production, poultry in a state of heat stress will take the initiative to reduce voluntary feed intake (Nawaz et al., 2021; Teyssier et al., 2022). Chronic heat stress impairs hypothalamic integrity and downregulates NPY mRNA and protein expression in broiler chickens, thereby suppressing appetite (He et al., 2019). Studies have demonstrated that dietary supplementation with a combination of 0.2% betaine and 0.79% glycine can effectively alleviate the decrease in feed intake caused by heat stress in Ross 308 broilers, resulting in a 10.61% increase in total feed intake compared to the heat-stressed group (Won et al., 2023), while the addition of 0.005% *Artemisia annua* oil and 1% *Artemisia annua* powder significantly increased the ADFI of heat-stressed broilers by 12.4% (Saracila et al., 2018). However, compared with a single nutritional intervention, environmental modifications, such as evaporative cooling pads and ventilation fans, are more effective at mitigating the root cause of appetite suppression (Mangan and Siwek, 2023).

Also, the needs of nutrition show clear differences in developmental stages (e.g., chick–rearing phase and laying phase). For instance, the starter phase needs high–protein and high–energy diets for the fast growing (Nahashon et al., 2006). Besides the changing of dietary formulation, supplemental complex enzyme preparation can also improve protein digestibility in the chicks (Kwak et al., 2024). Conversely, sufficient calcium and phosphorus should be ingested in the laying phase to maintain eggshell quality and laying rate (Garcia-Mejia et al., 2024). Dietary phosphorus deficiency in laying hens is correlated with reduced eggshell strength and decreased egg production (Mashaly et al., 2004; Tischler et al., 2013), and calcium will also damage the quality of shells (Fu et al., 2024). Therefore, it is beneficial to make the dietary nutrient profile according to the developmental stages and the production goal to optimise the practical goal.

The feeding regulation of different livestock breeds must match their unique physiological characteristics and the specific needs of each growth stage. In pig production, the risk of pollution that may be caused by mineral additives can be effectively reduced by implementing phased nutrition strategies and using enzyme preparations and organic acid substitutes. At the same time, the precise feeding system can dynamically adjust the level of amino acids according to individual needs. For ruminants, rumen health interventions include supplementing vitamin B_12_ in the perinatal period to prevent ketosis, optimizing the diet structure of sheep, and adopting a night feeding strategy to increase DMI. In the poultry breeding system, the comprehensive strategy of combining nutrition regulation with environmental control is crucial to alleviating heat stress. Starter diets require nutrient–dense formulations, while the laying phase necessitates calcium–phosphorus supplementation to ensure that feed collection and production performance are synchronized at a specific stage.

## 5 Research gaps and future directions

### 5.1 Novel protein sources and emerging strategies for feed intake regulation in livestock and poultry

In addition to exploring the regulating effect of traditional nutritional strategies such as probiotics, prebiotics, and phytogenic additives on the appetite of livestock and poultry, the research horizon is gradually expanding to more sustainable new feed resources. In recent years, high–protein substitute ingredients, such as insect protein and microalgae, have attracted much attention because of their nutritional potential and environmental benefits. A study on broilers found that when locust meal completely replaced fish meal in the diet (accounting for approximately 10% inclusion), broiler feed intake decreased; however, the FCR improved by 32.5% (Amobi et al., 2021). Furthermore, feed additives derived from different insect species can yield similar effects. According to research by Sajjad et al. (2024) reported that replacing up to 12% of soybean meal in Ross 308 broiler diets with meal from *Spodoptera frugiperda* or black soldier fly larvae significantly improved FCR by 12.4% and 11.9%, respectively, and daily weight gain by 14.4% and 14.7%, respectively, without affecting feed intake. This finding provides strong empirical support for the use of insect protein to meet the nutritional challenges of animals.

Although new feed resources such as insect protein and microalgae have attracted much attention, the specific mechanism of regulating the appetite of livestock and poultry has not been fully clarified, resulting in significant differences in the actual application effect. In Ross 308 broiler diets, the replacement rate of soybean meal with black soldier fly larvae powder is negatively related to feed intake, and the high–level substitution (50% replacement rate) often reduces ADFI by 12.3% (Murawska et al., 2021). A similar pattern is observed with algae–based feed additives. Supplementing the diets of 43 to 44 wk–old White Leghorn laying hens with *Spirulina* (supplemented with 2.5 g/kg) increased feed intake ((117.8 vs. 110.6 g/d in the control)) (Salahuddin et al., 2024), while other studies reported no significant effect (Curabay et al., 2021), and supplementation with *Chlorella* even reduced feed intake (Halle et al., 2009). Notably, algae often suppress feed intake in ruminants. Supplementing 103 g/d of Alga 1.0 to Jersey cows significantly reduced the DMI by 13.3% (Colin et al., 2024). Furthermore, adding 0.25% red seaweed to ruminant diets reduced feed intake by 9.95% and feeding rate by 39.6% (Nyløy et al., 2023). In summary, although algae and insects have become research hotspots for alternative protein sources because of their high protein characteristics, the contradictions of existing experimental results suggest that we need to deeply analyse their potential mechanism of action to provide theoretical support for the development and application of subsequent products.

It is worth noting that recent research has begun to focus on the synergistic effects of multiple additives. The study by Choudhury et al. (2021) demonstrated that a composite additive comprising various fibre sources, such as beet pulp and inulin, significantly increased feeding time in piglets before weaning by promoting gut microbiota maturation. Furthermore, the inclusion of a combination of four different additives (garlic powder at 0.3%, turmeric powder at 0.75%, yeast extract at 0.5%, and *parsilact* probiotic at 1.65%) in broiler chickens of Ross 308 diets does not significantly change the amount of food, but the effect of improving the FCR is better than that of a single additive, with an improvement ranging from 1.36% to 5.23% (Bozorgkhoo and Darmani, 2025). This finding suggests that certain combinations of additives, while not directly stimulating “appetite” (i.e., feed intake), can still significantly enhance nutrient utilisation efficiency, offering a broader perspective on appetite regulation. Therefore, future nutritional strategies targeting appetite may shift from merely stimulating intake towards an integrated intervention model of “appetite stimulation and efficiency promotion”. This also means that the development of insect protein and microalgae feed additives may be combined with conventional additives such as probiotics and prebiotics to give full play to their respective advantages through synergy.

### 5.2 Precision feed intake regulation via multi-omics and feed-focused genome editing

The rapid development of molecular biology and genomics technologies has brought new opportunities to the field of animal nutrition regulation. By deeply analysing the metabolic pathway and regulation mechanism of nutrients in animals, it is possible to clarify the molecular basis of appetite regulation more accurately, thus providing a new perspective for nutritional intervention. For example, using microbiome and metabolomics techniques, Wang et al. (2024) found that Duroc pigs with low residual feed intake (high feed efficiency) had significant enrichment of the gut microbiota, including *Clostridium_XVIII*, along with pronounced upregulation of the tryptophan metabolic pathway, especially for serotonin synthesis. This microbe-metabolite alteration is closely associated with specific phenotypic improvements: in extreme low–residual feed intake (RFI) individuals, ADFI decreased by approximately 21.0% compared to extreme high–RFI individuals, while ADG remained unaffected, ultimately resulting in an approximately 18.6% improvement in the FCR. This research suggests that microbial populations regulate satiety by enhancing the tryptophan-serotonin metabolic axis, thereby reducing feed intake and improving feed utilization efficiency. utilised plasma metabolomics to identify that pyridoxamine (a form of vitamin B_6_) in Duroc pig plasma is a key metabolite, which is significantly related to the residual feed intake, indicating its potential involvement in regulating feed efficiency. It is worth noting that vitamin B_6_ is an essential coenzyme in the process of transforming tryptophan into serotonin. Together, these two studies reveal the key role of the tight interaction between gut microbiota and host metabolism (e.g., the tryptophan–serotonin pathway) in regulating feed efficiency. This provides a new direction for livestock and poultry nutrition research: to systematically analyse the functional relationship between microorganisms, metabolites and feeding behaviour by using multi–omics technology, and then to formulate accurate nutrition regulation strategies based on the host**–**microbiota co–metabolic network.

Beyond metabolomics, genomics technologies can uncover the potential genetic basis for specific appetite preferences in local breeds. Comparative genomic analysis by Yang et al. (2024a) revealed significant differences in copy number variations (CNVs) and selection signals between Ningxiang pigs and Duroc pigs. The genetic variation of Ningxiang pigs mainly focuses on fat metabolism, digestion, and absorption. It is worth noting that the selected genomic regions are particularly related to the pathway of carbohydrate digestion and absorption, which provides a genetic explanation for their roughage tolerance. Despite the increasing maturity of gene–editing technologies, such as clustered regularly interspaced short palindromic repeats associated protein 9 (CRISPR–Cas9), and their remarkable progress in research on species disease resistance (Xu et al., 2020), it is still challenging to apply this technology to regulate basic behaviours like feed intake. Although potential gene editing targets have been identified, for instance, El-Magd et al. (2016) reported that single nucleotide polymorphisms (SNPs) in the growth hormone secretagogue receptor gene (*GHSR*) significantly enhance appetite in poultry; specifically, in Mandara chickens, individuals with the GG/GG *GHSR* haplotype exhibited significantly higher feed intake than AG/AG individuals from 4 to 8 wk of age (an increase of 61.4 g). However, feed intake, as a highly complex quantitative trait, is regulated by numerous minor–effect genes (Yang et al., 2011; Yuan et al., 2015). Therefore, in the absence of sufficient verification of causal mutations and their pleiotropic effects, direct editing of appetite-related genes may lead to unpredictable adverse consequences, thus limiting the recent application prospect of gene editing in this field.

Moreover, an emerging perspective involves genetically editing plant–derived feed to improve the nutritional status of livestock and poultry. For example, genome editing technologies have been successfully employed in various staple crops, including rice, wheat, maize, and potato, to reduce antinutritional factors (e.g., phytic acid and alkaloids) or enhance the contents of nutritional components (e.g., proteins, vitamins, and functional metabolites) (Jansman et al., 2010; Ku and Ha, 2020; Zhang et al., 2024). Furthermore, soybeans, a major protein source in animal feed, contain anti–nutritional factors that impair the digestion, absorption, and utilisation of nutrients (Hong et al., 2004). Miller et al. (2025) demonstrated that trypsin inhibitors significantly reduced ADFI in nursery pigs. Therefore, gene editing technology can be used to reduce the content of trypsin inhibitors in soybean. The Kunitz trypsin inhibitor (KTI) is the main anti-nutritional factor in soybean, which seriously affects the digestion and absorption of protein. Researchers have successfully used this technology to knock out KTI–related coding genes and created an improved soybean strain with extremely low or even undetectable trypsin inhibitor activity (Wang et al., 2022). Compared with the complexity and pleiotropic risk of directly editing animal appetite–related genes, focusing gene–editing technology on improving the plant feed ingredient is a safer, more feasible strategy to indirectly regulate feed intake and to improve feed efficiency.

In summary, the cooperation of omics analysis and gene editing technology is continuously expanding our knowledge of the feeding regulation mechanism of domestic animals and poultry. In the future, with the help of a multi–omics strategy, researchers are expected to reveal the process of appetite regulation more systematically and deeply analyse the common metabolic network formed between host sources and dietary sources. When these strategies are combined with accurate genome editing technology to improve the nutritional characteristics of feed components, it will help to establish nutritional intervention measures based on the action mechanism, so as to regulate the feeding behaviour of livestock and poultry more efficiently and accurately.

### 5.3 Application potential and implementation challenges of precision feeding systems

Precision feeding, as a novel nutritional strategy for livestock and poultry, dynamically adjusts feed formulation and allocation based on real–time data and individual animal variations, aiming to precisely meet nutritional requirements and optimise production efficiency. Precision feeding systems have been widely adopted across various livestock and poultry production systems, although their application has primarily focused on data collection (Aquilani et al., 2022). In terms of practical intervention, such equipment currently focuses more on indirectly improving feed efficiency through optimised feeding management. Specifically, by fine–tuning the dietary composition for livestock and poultry, precision feeding can regulate nutrient intake to enhance feed utilisation efficiency in livestock and poultry (Andretta et al., 2014).

For instance, a study by Efendiev et al. (2024) found that in a commercial poultry house rearing approximately 27,000 Cobb 500 broilers, the implementation of a fully automated system reduced the FCR by 13.9% (from 1.838 to 1.582 kg feed/kg gain) and increased the ADG by 22.3%. Over a 42**-**d cycle, this resulted in about 105,000 rubles in electricity cost savings per house and generated nearly 1 million rubles in additional revenue due to a 23.9% increase in output.

However, precision feeding systems rely heavily on a suite of sensing and automation technologies to enable individual management, including automatic feeding control units, feeding behaviour monitoring systems, and radio frequency identification (RFID)–based individual identification (Rathod and Dixit, 2020). The cost of equipment capable of managing only 20 to 25 pigs is as high as 1300 to 2000 euros. Since these fixed costs are not easy to amortise, this kind of system is not economically feasible for small–scale producers (Pardo et al., 2022). Moreover, producers used to traditional husbandry practices will have big problems with the new technical and managerial demands that are introduced with precision feeding systems (Ait-Sidhoum et al., 2021).

To reduce the deployment cost, optimization algorithms and monitoring methods have emerged as potential alternatives. For example, by monitoring the heart rate and activity, the net energy requirement of pigs can be effectively estimated (Li et al., 2024b), and an artificial neural network model also shows good effect in predicting the individual net energy requirement (Yang et al., 2024c). Additionally, the precision feeding system is an efficient delivery platform for feed additives, and the prospect of improving the feed intake of livestock and poultry is increasingly broad. For instance, Hanson (2023) employed the SmartFeed Pro system to deliver pellets containing a caramel flavouring agent to beef cattle. While this did not significantly shorten the adaptation period or improve the accuracy and consistency of feed intake, it validated the feasibility of using an individualised, quantifiably controlled hardware platform to evaluate the impact of functional additives on feeding behaviour. Consequently, future research can focus on reducing the implementation cost of a precision feeding system and exploring its application potential as a personalized feed additive management platform, aiming at effectively improving the feed intake of livestock and poultry under different health or environmental conditions.

## 6 Conclusions

Regulation of appetite in livestock and poultry is controlled by complex interactions among central neural circuits, peripheral signals, and the environment, where nutrition is a major regulator. In this review, we summarise and assess the roles of various nutritional factors and new technologies in appetite modulation, and point out existing gaps in the exploration of the mechanisms and industrial applications. To connect the theory with practice, we make the following four recommendations: (1) implement species–and stage–specific nutritional protocols for swine, ruminants, and poultry; (2) develop safe composite functional additives based on the gut–brain axis; (3) promote gradient precision feeding systems tailored to farm scales; (4) integrate nutritional interventions with environmental management to alleviate stress-induced inappetence. Future research should further interdisciplinary collaboration to translate the new knowledge from multi–omics and gene–editing into low-cost and adaptable strategies to help animal husbandry to achieve sustainable and precise regulation of appetite.

## Credit Author Statement

**Xiangyan Liu:** Writing – original draft, Conceptualization. **Ruxin Yao:** Writing – original draft. **Shiyu Song:** Writing – original draft. **Chuan Zhou:** Writing – review & editing. **Lixin Wen:** Funding acquisition, Conceptualization. **Ji Wang:** Writing – review & editing, Conceptualization.

## Declaration of conflict of interests

We declare that we have no financial and personal relationships with other people or organizations that can inappropriately influence our work, and there is no professional or other personal interest of any nature or kind in any product, service and/or company that could be construed as influencing the content of this paper.
